# The role of health facility and individual level characteristics on medication adherence among PLHIV on second-line antiretroviral therapy in Northeast Ethiopia: use of multi-level model

**DOI:** 10.1186/s12981-022-00441-8

**Published:** 2022-03-26

**Authors:** Shambel Wedajo, Getu Degu, Amare Deribew, Fentie Ambaw

**Affiliations:** 1 School of Public Health, CMHS, Wollo University, Dessie, Ethiopia; 2 School of Public Health, CMHS, Bahir Dar University, Bahir Dar, Ethiopia; 3 School of Public Health, St. Paul Hospital Millennium Medical College, Addis Ababa, Ethiopia

**Keywords:** Second-line ART, Adherence, Facility-level determinants, HIV/AIDS, Ethiopia

## Abstract

**Background:**

Medication adherence plays a pivotal role in achieving the desired treatment outcomes. The proportion of HIV patients on second-line antiretroviral therapy is becoming a growing public health concern. However, to date, little attention has been given to second-line antiretroviral medication adherence. Moreover, the association between health facility characteristics and medication adherence has yet not been tested. Thus, this research was conducted to determine the magnitude of medication adherence and examine the role of facility-level determinants among HIV patients on second-line ART.

**Methods:**

A cross-sectional study was conducted on 714 HIV patients on second-line therapy who were selected via systematic random sampling in twenty public health facilities. Medication adherence was measured using the six-item Simplified Medication Adherence Questionnaire (SMAQ) tool. Data were collected in a personal interview as well as document reviews. A multi-level binary logistic regression was used to uncover individual and facility-level determinants. The effect size was presented using an adjusted odds ratio (AOR), and statistical significance was declared at a P value less than 0.05.

**Results:**

The magnitude of optimal medication adherence among HIV patients on second-line antiretroviral therapy was 69.5% (65.9–72.7%). Medication adherence was positively associated with the use of adherence reminder methods [AOR = 3.37, (95% CI 2.03–5.62)], having social support [AOR = 1.11, (95% CI 1.02–1.23)], and not having clinical depression [AOR = 3.19, (95% CI 1.93–5.27). The number of adherence counselors [AOR = 1.20, (95% CI 1.04–1.40)], teamwork for enhanced adherence support [AOR = 1.82, (95% CI 1.01–3.42)], and caseloads at ART clinics were all significantly correlated with ARV medication adherence at the facility level.

**Conclusions:**

A large proportion of HIV patients on second-line antiretroviral therapy had adherence problems. Both facility-level and individual-level were linked with patient medication adherence. Thus, based on the identified factors, individual and system-level interventions should be targeted.

## Introduction

Despite the availability of effective antiretroviral therapy, medication adherence is critical for achieving the desired therapeutic outcomes, which necessitates taking 95% and more of their prescribed drugs [1, 2]. However, poor medication adherence is the leading cause of first-line antiretroviral treatment failure as shown in various studies [3–5]. Poor antiretroviral medication adherence has multidimensional consequences, at the individual, public, and policy levels. Individually, it leads to uncontrolled viral load [6–8], increases the risk of drug resistance [9], treatment failure [10], and compromises the overall quality of life, including death. It also raises the risk of HIV transmission and incurs high health care costs at the public level [11]. Furthermore, it jeopardizes the 95%, 95%, 95% UNAIDS fast track targets at policy-level [12, 13].

Currently, the number of HIV patients on second-line antiretroviral therapy is becoming a growing public health concern. A considerable number of patients worldwide have experienced treatment failure and switched to second-line antiretroviral therapy [14]. In Ethiopia, the proportion of people living with HIV (PLHIV) on second-line antiretroviral therapy is also rising; according to a systematic review, 15.9% (11.6–20.1%) of PLHIV had failed their first-line treatment [3] and switched to second-line antiretroviral therapy. However, unlike patients on first-line therapy, to date, little is known about medication adherence in HIV patients who are taking second-line antiretroviral therapy. First and second-line antiretroviral therapies vary in drug formulation, palatability, and possible side effects, besides the nature of the regimen [1]. If the patient does not respond to second-line therapy, he or she will be switched to high-cost third-line ARV, which is the patient’s last treatment option or salvage therapy [2]. Hence, to prolong the use and efficacy of second-line therapy and to prevent further treatment failure, assessing adherence issues in such patients is so crucial. Adherence is a behavior and a dynamic that is influenced by circumstances. Hence, assessing adherence and its contextual determinants is a continual process.

Furthermore, most previous studies have focused on individual-level determinants and failed to examine the role of health facility characteristics on patient medication adherence [15–18]. To the best of our knowledge, no research has been done in the Ethiopian health system context to assess the impact of individual-level and health facility characteristics on medication adherence for PLHIV on second-line ART. Generating local evidence is required for context-based decision making, and allows for the crafting of appropriate interventions that enhance patient medication adherence. Some of the health facility characteristics assumed to affect medication adherence include PLHIV caseloads at ART clinics, drug stock outs, average time for one-on-one counseling, number of healthcare providers and their average experience, number of adherence counselors, and other facility characteristics were considered [10, 16, 19–22].

Hence, based on the above-identified gaps, we conducted a cross-sectional study using a multi-level model of analysis to determine the magnitude of medication adherence and to investigate the association between medication adherence and facility-level characteristics among PLHIV on second-second antiretroviral therapy.

## Methods

### Study setting

This study was carried out in public health facilities in the Eastern Amhara region of Northeast Ethiopia that currently provide second-line antiretroviral therapy. According to Ethiopia’s national HIV/AIDS policy, hospitals and health centers (having more than 200 HIV cases) are authorized to commence second-line antiretroviral treatment. Eastern Amhara is a high-burden area in the region [23, 24], and 2332 PLHIV are currently receiving second-line antiretroviral therapy.

In Ethiopia, standard second-line antiretroviral therapy consists of a combination of three ARV drugs (at least two of which are new to the patient); two Nucleoside Reverse Transcriptase Inhibitors (NRTIs) as a backbone; Lamivudine (3TC) and Abacavir (ABC), or Zidovudine (ZDV) or Tenofovir (TDF) and one protease Inhibitors (PIs); Lopinavir/ritonavir (LPV/r) or Atazanavir/ritonavir (ATV/r) [2].

Moreover, all facilities use the same documentation and reporting system, and HIV data are handled by SMART care, the ART logbook, and the chronic ART follow-up form [2]. These registries are updated after each clinical appointment. Currently, HIV patients are scheduled every three months. Patients will be assessed for nutritional status, opportunistic infections, medication adherence, drug side effects, and refilled with ART and other preventative medications at each appointment. Antiretroviral treatment success is monitored using clinical, immunological, and viral load (VL) assessment, in which a viral load test is done after initiation of ART at 6 months, 12 months, and every 12 months and serves as a gold standard monitoring tool [1, 2].

#### Study design and period

A cross-sectional study was conducted in the Eastern Amhara region, Northeast Ethiopia from December 2020 to February 2021.

#### Study population

Adult people living with HIV who were taking second-line antiretroviral therapy in Eastern Amhara region during the data collection period were considered as the study population.

#### Sample size

A sample of 719 HIV patients on second-line therapy was determined using 7.2.2.6 EPI Info software package by taking 69.3% proportion of optimal medication adherence to second-line antiretroviral therapy from a study done in Tanzania [10], 95% confidence level, and 5% margin of error, with a design effect of two, and 10% non-response rate.

#### Sampling procedures

The sample individuals were selected by using a systematic random sampling method in twenty public health facilities. Of those facilities, six were hospitals, and fourteen were health centers. First, the sampling frame was secured in each selected facility by reviewing the updated ART registration logbook. Then, based on the number of patient loads, samples were proportionally allocated to each health facility. Finally, using the systematic random sampling method, sample clients were recruited in every third interval while the clients came for routine follow-up.

### Variables and measurement

#### Outcome variable

Second-line antiretroviral medication adherence which was measured using six-item Simplified Medication Adherence Questionnaire (SMAQ) tools [25]. The six SMAQ includes: “1. Do you ever forget to take your medicine?”, “2. Are you careless at times about taking your medicine?”, “3. Sometimes if you feel worse, do you stop taking your medicines?”, “4. Thinking about the last week, how often have you not taken your medicine?”, “5. Did you not take any of your medicine over the past weekend?”, and, “6. Over the past 3 months, how many days have you not taken any medicine at all?” When a patient answers “no” to questions 1, 2, 3, and 5, zero for question 4, and any response less than 2 for question 6, she or he was considered to be optimal adherence. This six-item SMAQ scale was further validated in HIV patients in Ethiopia [26]. In this study, the internal consistency of the SMAQ tool was also assessed and found 0.87 Cronbach alpha value.

#### Individual-level factors

Age, sex, residency, marital status, religion, education status, duration on ART, disclosure, use of adherence reminder methods, substance use, depression, social support, and independent source of income were among the socio-demographic and behavioral factors. Body mass index (BMI), functional status, viral load status, CD4 cells/mm^3^, WHO-clinical stage, drug substitution history, reported drug side effects, and comorbidity were all clinical variables. Comorbidity status of an individual assessed using a self-report method. A patient who had one or more of the following confirmed non-communicable illnesses (NCDs); diabetes, cardiovascular disease, hypertension, and cancer were considered as co-morbid cases. Viral load status is classified as high and low viral load. A patient having last viral load status greater or equal to 1000 copies/mL is classified as high viral, unless considered as low viral load [1, 2].

Depression symptoms were measured using a nine-item Patient Health Questionnaire (PHQ-9) [27]. The total score ranges from 0 to 27. Patients having depression scores of ten and above were taken as having clinical depression. This PHQ-9 items depression screening tool was validated in the Ethiopian context [28]. Social support was measured using the three**-**item Oslo Scale [29]. The Oslo Scale’s total score ranges from 3 to 14 and is categorized as “poor support” (3–8), “moderate support” (9–11), and “strong support” (12–14). The risk of substance use was measured using the WHO Alcohol, Smoking, and Substance Involvement Screening Test (ASSIST) (V.3.1), which consists of seven items for each of alcohol, khat use, and tobacco products [30]. Participants were then classified as having low, moderate, or high-risk substance use based on the cut-point of WHO-ASSIST scoring.

Functional status is defined as an individual’s ability to perform normal daily activities required to meet basic needs and fulfill the usual working activities. It was classified as follow; “workable functional status” or “W”: able to perform the usual work activity, “ambulatory” or “A”: able to perform the activity of daily living, but not able to work, and “bedridden” or “B”: not able to perform the activity of daily living. In this study, ambulatory and bedridden participants were categorized as not having workable functional status [1, 2].

Two criteria were used to classify CD4 cells. Healthy individual CD4 cell counts range from 450 to 1500. The risk of opportunistic infection begins in HIV patients when CD4 cell counts are less than 450. Hence, based on the above two concepts, 450 cells/mm^3^ was used to categorize the last CD4 cell measurement [1, 2].

#### Facility-level factors

PLHIV caseload at ART clinics, reported second-line ARV drug stock out in the last six months, type of health facility, average time for one on one counseling, number of healthcare providers and their average experience, number of adherence counselors or supporters, the average proportion of perceived satisfaction on one on one counseling, the average perceived good patient-provider interaction, and teamwork in EAS intervention were all considered as health-care facility characteristics.

#### Data collection

Data were collected using a structured questionnaire via face-to-face interviews by trained nurses at the ART outpatient department (ART-OPD). A questionnaire was prepared in the local language, and data were collected for a 3-month duration while the patient came for a routine clinical visit. Moreover, document reviews were also done to extract patient clinical profiles. The extraction sheet was prepared as per the national consolidated antiretroviral guideline [2]. To ensure the quality of the data, the questionnaire was pretested, and data collectors were trained on data collection procedures. Moreover, onsite supervision was made by the assigned supervisors in each facility during data collection.

#### Data analysis

Data were cleaned and entered into EpiData Version 3.1 software. Then, exported to Stata version 14 (StataCorp, USA) for further analysis. For categorical variables, frequency (%) was computed. For a continuous variable, first distributional assumptions were checked using Kolmogorov–Smirnov and Shapiro–Wilk test. Then, for normally and skewed distributed continuous variables, mean with standard deviation (SD) and median (interquartile range, IQR) were employed, respectively.

After computing the Intra Class Correlation coefficient (ICC), which was 20.3%, a multi-level binary logistic regression was used to find out individual and facility-level determinants. The ICC measures the percentage of variation in medication adherence that can be explained by health facility characteristics, which are computed from an intercept-only model. ICC = $\tau oo/[\tau oo+\left(\frac{\pi 2}{3}\right)]$ [31, 32].

The final model was presented by computing the null model, individual-level only model, and combined model (i.e., individual and facility-level factors). The model with the smallest deviance was chosen as the best fit model using Akaike’s Information Criterion (AIC). The effect size was presented using an adjusted odds ratio (AOR), and statistical significance was declared at a P value less than 0.05.

## Results

### Socio-demographic and behavioral characteristics

Out of 719 approached sample HIV patients on second-line antiretroviral therapy, 714 (99.3%) patients agreed and gave their consent to participate in this study.

Of those surveyed, 468 (65.5%) lived in urban areas, 400 (56%) were female, and 342 (47.9%) were married. Concerning the source of income, 511 (71.6%) participants had an independent source of income. Similarly, of the 714 participants, 433 (60.6%) had attained formal education, and 355 (49.7%) were orthodox religious followers. Regarding social support and depression status, 366 (51.2%) and 516 (72.3%) of participants have moderate social support and no clinical depression, respectively.

Regarding utilization of adherence reminder tools, 612 (85.7%) of participants used adherence reminder methods such as phones, watches, and clock alarms. Of those surveyed, 574 (80.4%) participants were low-risk substance users. The median (IQR) age and year on antiretroviral therapy of participants were 37 (30–45) years and 10 (8–13) years, respectively (Table 1).

**Table 1:** Socio-demographic and behavioral characteristics of HIV patients on second-line antiretroviral therapy in Eastern Amhara region, Northeast Ethiopia, December 2020–February 2021 (n = 714)

Socio-demographic and behavioral characteristics	Total (714) N (%)	Medication adherence (496) N (%)
Sex		
Female	400 (56.0)	281 (70.2)
Male	314 (44.0)	215 (68.5)
Residence		
Urban	468 (65.5)	322 (68.8)
Rural	246 (34.5)	174 (70.7)
Marital status		
Married	342 (47.9)	246 (71.9)
Not married	372 (52.1)	250 (67.2)
Religion		
Orthodox	355 (49.7)	239 (67.3)
Muslim	346 (48.5)	248 (71.7)
Protestant	10 (1.4)	6 (60.0)
Catholic	3 (0.4)	3 (100.0)
Educational status		
Formally educated	433 (60.6)	305 (70.4)
Not formally educated	281 (39.4)	191 (68.0)
Independent source of income		
Yes	511 (71.6)	371 (72.6)
No	203 (28.4)	125 (61.6)
Social support status		
Strong social support	171 (23.9)	139 (81.3)
Moderate social support	366 (51.2)	282 (77.0)
Poor social support	177 (24.9)	75 (42.4)
Risk of substance use		
Low-risk substance use	574 (80.4)	406 (70.7)
Moderate and severe risk substance use	140 (19.6)	90 (64.3)
Disclosure status		
Yes	654 (91.6)	460 (70.3)
No	60 (8.4)	36 (60.0)
Use of adherence reminder methods		
Yes	612 (85.7)	457 (74.7)
No	102 (14.3)	39 (38.2)
Depression status		
Not have clinical depression	516 (72.3)	411 (79.7)
Have clinically depression	198 (27.3)	85 (42.9)
Age, median (IQR) (year)	37 (30–45)	38 (31–45)
Year on ART, median (IQR) (year)	10 (8–13)	11 (8–13)

### Clinical characteristics

Out of 714 study participants, 545 (76.3%) had a BMI of ≥ 18.5 kg/m^2^, while 540 (75.6%) participants had viral load measurements below 1000 copies/mL. Similarly, 685 (95.9%) and 608 (85.2%) participants were not in advance of the WHO clinical stage and workable functional status, respectively. Regarding drug side effects and comorbidity status, 640 (89.6%) patients had not reported a drug side effect and 63 (8.8%) patients had confirmed comorbid diseases. TDF-3TC-ATV/r (361(50.6%)) and AZT-3TC-ATV/r (338(47.3%) were the most prescribed second-line antiretroviral regimens (Table 2).

**Table 2:** Clinical characteristics of HIV patients on second-line antiretroviral therapy in Eastern Amhara region, Northeast Ethiopia, December 2020–February 2021 (n = 714)

Clinical characteristics	Total (714) N (%)	Medication adherence (496) N (%)
BMI		
≥ 18.5 kg/m^2^	545 (76.3)	395 (72.5)
< 18.5 kg/m^2^	169 (23.7)	101 (59.8)
Functional status		
Workable	608 (85.2)	435 (71.5)
Not workable	106 (14.8)	61 (57.5)
WHO clinical stages		
I and II	685(95.9)	478(69.8)
III and IV	29 (4.1)	18 (62.1)
Last HIV viral load status		
High viral load (VL ≥ 1000copies/mL)	174 (24.4)	91 (52.3)
Low viral load (VL < 1000 copies/mL)	540 (75.6)	405 (75.0)
Last CD4 cells measurement		
≤ 450 cell/mm^3^	603 (84.5)	412 (68.3)
> 450 cell/mm^3^	111 (15.5)	84 (75.7)
Drug substitution history		
No	505 (70.7)	361 (71.5)
Yes	209 (29.3)	135 (64.6)
Reported side effect		
No	640 (89.6)	451 (70.5)
Yes	74 (10.4)	45 (60.8)
Comorbidities status		
No	651 (91.2)	457 (70.2)
Yes	63 (8.8)	39 (61.9)
Second-line ART		
TDF—3TC—ATV/r	361 (50.6)	259 (71.7)
AZT—3TC—ATV/r	338 (47.3)	230 (68.0)
TDF—3TC—LPV/r	11 (1.5)	4 (36.4)
AZT—3TC—LPV/r	4 (0.6)	3 (75)

*BMI* body mass index; *TDF* tenofovir; *3TC* lamivudine; *ATV/r* atazanavir/ritonavir; *AZT* zidovudine; *LPV/r* lopinavir/ritonavir; *ART* antiretroviral therapy

### Facility-level characteristics

Five out of twenty health facilities reported having second-line ARV drug stock out in the last six months. In nine out of twenty health facilities, a team of health professionals and adherence support delivered the Enhanced Adherence Support (EAS) intervention to high viral load patients. The median (IQR) PLHIV caseloads and the number of health care providers at ART clinics were 1569 (1069–2069) and 3 (2–4.75), respectively. Similarly, the median (IQR) number of adherence counselors and one-on-one counseling time were 3.5 (2–4.75) and 7.5 (5–9.4), respectively (Table 3).

**Table 3:** Health facility characteristics in Eastern Amhara region, Northeast Ethiopia, December 2020–February 2021 (n = 20)

Facility characteristics	Summarization
PLHIV-caseloads, median (IQR)	1569 (1069–2069)
Number healthcare providers who provide ART service, median (IQR)	3 (2–4.75)
Average experience of healthcare providers, mean (SD)	5.57 ± 2.18
Number of adherence counselors, median (IQR)	3.5 (2–4.75)
Time spent for one-on-one counseling, median (IQR), minute	7.5 (5–9.4)
Average proportion of perceived satisfaction on counseling	0.74 ± 0.2
Average perceived good patient-provider interaction	0.72 ± 0.24
Reported second-line ARV drug stock out in the last 6 months	5 (25%)
Who provides Enhanced Adherence Support (EAS) intervention?	
Adherence supporter alone	8 (40%)
Health professional alone	3 (15%)
Both as a team	9 (45)

### Factors associated medication adherence

In this study, the magnitude of medication adherence among HIV patients who were taking second-line antiretroviral therapy was 69.5% (65.9–72.7%). Individual-level characteristics that were positively associated with medication adherence included the use of adherence reminders, having social support, and not having clinically significant depression symptoms. Similarly, at the facility level, PLHIV caseloads at ART clinic, the number of adherence counselors or supporters, and implementation of EAS by teamwork were all substantially correlated with medication adherence.

When a patient’s social support score increased by one unit, the odds of sticking to their therapy on average improved by 11% while holding the effect of other covariates in the model remained constant [AOR = 1.11, (95% CI 1.02–1.23)]. Similarly, patients who did not have clinically significant depression symptoms were more likely to adhere to their antiretroviral medication than their counterparts [AOR = 3.19, 95% CI 1.93–5.27]. Patients who had been using adherence reminder methods were more likely than their counterparts to adhere to their treatment [AOR = 3.37, (95% CI 2.03–5.62)].

The odds of patients adhering to their medication improved on average by 20% for each increment of adherence counselors in the facility, while all other variables in the model remained constant [AOR = 1.20, (95% CI 1.04–1.40)]. The odds of patients adhering to their therapy were higher in a facility where EAS was given by the team [AOR = 1.82, (95% CI 1.01–3.42)]. For every increase in the number of PLHIV cases in a facility, the likelihood of patients adhering to their medication decreased by 0.3 percent [AOR = 0.997, (95% CI 0.995–0.999)], (Table 4).

**Table 4:** Multilevel binary logistic regression model on determinants of medication adherence among HIV patients on second-line antiretroviral therapy in Eastern Amhara region, Northeast Ethiopia, December 2020–February 2021 (n = 714)

Factors	Intercept only model AOR, (95% CI	Individual-level factor only model AOR, (95% CI)	Facility and individual level combined model AOR, (95% CI)
	2.29		
Use of adherence reminder methods: yes		3.70 (2.22–6.17)	3.37 (2.03–5.62)
Perceived social support		1.14 (1.04–1.25)	1.11 (1.02–1.23)
Depression: not having clinical depression		3.43 (2.08–5.67)	3.19 (1.93–5.27)
Number of adherence counselors			1.20 (1.04–1.40)
Teamwork for EAS intervention			1.82 (1.01–3.42)
PLHIV-Caseload in the facilities			0.997 (0.995–0 0.999)

The findings were after controlling variables including, age, sex, disclosure status, WHO-clinical staging, drug side effect, drug stakeouts, and average time in one on one counseling

## Discussion

This study aimed to determine the magnitude of second-line antiretroviral medication adherence and to test the association between health facility characteristics and medication adherence. Seven out of ten HIV patients were compliant with the prescribed antiretroviral medication. Aside from individual-level factors, the identities of healthcare facilities were substantially linked to antiretroviral medication adherence.

In this study, 69.5% of HIV patients who were taking second-line antiretroviral therapy had optimal medication adherence. This finding is consistent with other [10, 16–18, 33, 34]. However, it falls short of the national and WHO requirements of 95% or greater adherence [1, 2]. Most first-line treatment failures were associated with poor adherence [3–5]. Such patients might be switched to next-level therapy without fully addressing adherence barriers, which compromise the compliance of subsequent therapy [10]. Consequently, second-line medication adherence might be lower than expected. Furthermore, being on second-line therapy and the nature of the regimen; have no fixed-dose combination [35, 36], may also contribute to the lower proportion of adherence in patients on second-line therapy. In general, this result suggests that second-line medication adherence is not as per standard. A considerable number of patients are at risk of further treatment failure, hence special attention should be paid to them. Alternative adherence intervention packages should be bolstered, including prolonging EAS intervention after the commencement of second-line therapy.

Medication adherence was positively associated with social support, which is in agreement with other studies [18, 37–41]. Patients who receive social support from family and friends may have a stronger sense of self-worth, which might help them to be more optimistic about their therapy [42]. Furthermore, support from family and friends should be encouraged at the societal level, as it is both cost-effective and enables patient-centered intervention. In addition to health education and counseling at health facilities, support from social support should also be encouraged. Social support helps patients to acquire strength, courage and allows them to feel safe and supported during their illness [43], which further improves therapy compliance.

Medication adherence was inversely associated with the severity of depression symptoms. This finding is consistent with other studies [34, 44–50]. Demotivation, social withdrawal, and hopelessness can all impair treatment adherence. Depression impairs one’s ability to think, reason, and self-management ability in general. Thus, screening, prevention, and control strategies including psychosocial support should be strengthened in routine clinical care by integrating with WHO’s mental health Gap Action Programme (mhGAP) [51].

The use of adherence reminders was positively associated with mediation adherence, which is supported by other studies [33, 52, 53]. Reminders might serve as a memory aid that encourages patients to take their medications on a more regular basis. Hence, context and individual-based adherence reminder methods should be promoted during HIV counseling as an important strategy to improve antiretroviral adherence.

HIV caseload at the ART clinic was inversely associated with medication adherence. Aside from the clinical review, patients with HIV require comprehensive HIV care and treatment, including reviewing psychosocial, cognitive, behavioral, emotional, and socio-economic barriers to enhance medication adherence. However, in high caseload facilities, healthcare providers and adherence supporters might fail to assess the potential barriers to medication adherence. Workload should be balanced in order to provide quality health care services [54]. Hence, increasing decentralization of existing facility-based ART services as well as customizing new community-based ART delivery modalities [55] including community ART distribution points, community ART groups (CAGs), and community-based adherence clubs, should be promoted to lessen the burden of caseload.

Similarly, the number of adherence counselors or supporters was positively correlated with antiretroviral medication adherence. This finding is in agreement with a multi-level study done in Cambodia [19]. Adherence supporters are formally assigned non-healthcare workers who provide adherence counseling, screening for mental health and substance use problems, trace loss to follow-up cases, and provide patient center one-on-one counseling or mass education about treatment and illness [2, 56]. Thus, to provide effective adherence counseling and other jobs, the number of adherence supporters should be increased in tandem with the number of patients.

Enhanced adherence support (EAS) is given to PLHIV patients who have an uncontrolled viral load (viral load > 1000 copies/mL), clinical deterioration, persistence immunosuppression, or multiple adherence barriers [2]. Our study revealed that patient medication adherence was lower in facilities where EAS was not implemented in a team. Health professionals and adherence supporters are expected to work together to improve EAS. This allows us to review and correct cognitive, behavioral, emotional, and clinical, drug-related side effects, and socioeconomic adherence hurdles from different perspectives. Consequently, the likelihood of having adherence problems might be lower in such facilities as compared with facilities that have no teamwork during EAS intervention. A healthcare team does have the capacity to achieve more than any individual could do alone [57].

## Conclusions

A high number of HIV patients on second-line antiretroviral therapy had adherence problems. Health facility characteristics; caseloads, number of adherence counselors, and teamwork were strongly linked with medication adherence. Thus, system-level intervention should be undertaken besides individual-level intervention to improve medication adherence, so as prolog the use of second-line therapy, and prevent further treatment failure.

### Strength of the study

First, the study is the first of its kind in Ethiopia to provide first-hand information on the magnitude of second-line antiretroviral medication adherence. Second, this study examines the role of health facility characteristics besides individual-level determinants using multi-level analysis. Third, it was conducted with a large sample size and the findings can be generalized to other low-income settings where similar WHO HIV treatment modalities are used.

### Limitation of the study

In a cross-sectional design, it was difficult to ascertain causal inferences between the outcome variable (medication adherence) and the explanatory variables like depression status and clinical stages. Hence, care should be taken for temporality while interpreting the findings of this study, and longitudinal research should be considered as a possible solution that allows the evaluation of adherence episodes across time after the commencement of ARV. Besides, medication adherence was assessed using the self-report method, which is an indirect method that is prone to overestimation of adherence. Further, Alcohol, Smoking, and Substance Involvement Screening Test (ASSIST) (V.3.1) tool, which used in this study was not validated in the Ethiopia.

## Data Availability

The materials, datasets used and analyzed in the current study are available from the corresponding author upon reasonable request.

## Abbreviations

SMAQ: Simplified Medication Adherence Questionnaire
ART: Antiretroviral therapy
PLHIV: People living with HIV
VL: Viral load
BMI: Body Mass Index
IQR: Interquartile range
PHQ-9: Nine Patient Health Questionnaire
NCD: Non-communicable disease (NCDs)
EAS: Enhanced adherence support
